# Moderate dose melatonin for the abatement and treatment of delirium in elderly general medical inpatients: study protocol of a placebo controlled, randomised, double blind trial

**DOI:** 10.1186/s12877-016-0230-5

**Published:** 2016-02-29

**Authors:** Daniel I. Clayton-Chubb, Peter W. Lange

**Affiliations:** Alfred Health, Melbourne, VIC Australia; Department of Geriatrics, Royal Melbourne Hospital, Parkville, VIC Australia; Faculty of Medicine, Dentistry and Health Sciences, University of Melbourne, Parkville, VIC Australia

## Abstract

**Background:**

Delirium is a frequent, costly and morbid problem. No agent has been shown to modify the natural history of the condition, and current treatments have significant side effects. Prophylactic melatonin in low doses has been shown to prevent delirium developing. This trial then aims to determine the feasibility of a trial to assess if melatonin at a moderate dose effectively treats the symptoms of delirium and modifies the natural history, including abating symptoms after treatment cessation.

**Methods/design:**

Elderly (≥70 years of age) patients admitted to the Royal Melbourne Hospital with delirium, and not requiring surgery, will be identified from the current practice of the investigators and through referral by other general medical unit staff. To facilitate this, other staff will be briefed on the project by investigators. Patients will be recruited with suitable informed and documented consent (person responsible) by the study investigators. They will receive orally either 5 mg melatonin (18 patients) or placebo (18 patients) nightly for 5 nights (or until discharged).

During treatment, participants will be assessed by study staff using a validated scale of delirium severity (the Memorial Delirium Assessment Scale), and a validated measure of delirium state (Confusion Assessment Method) to determine if melatonin decreases the severity or the duration of delirium. Assessment will continue for a further two days after treatment has ceased, to determine if the treatment causes persisting abatement of symptoms, and to assess for adverse events.

**Discussion:**

The on-going study described herein will contribute to our knowledge of available treatment options for elderly inpatients with delirium, where current pharmacological interventions show weak or no effect on hastening the resolution of delirium. As melatonin is safe, cheap, and potentially effective, it would be easily implementable in routine practice and could lead to significant outcome benefits for delirious inpatients.

**Trial registration:**

The trial is registered with the Australia New Zealand Clinical Trials Registry (trial ID: ACTRN12614000101684) (registered 28/01/2014).

## Background

Delirium is a clinical syndrome of acute onset characterised by fluctuating attention and awareness with a disturbance in cognition due to the direct physiological consequence of a medical condition, intoxication, withdrawal, toxin, or multiple aetiologies [1]. Melatonin (N-acetyl-5-methoxytryptamine) is a hormone (and likely neurotransmitter) produced by the pineal gland in the human brain. It is involved in sleep/wake cycle regulation. Delirium is a condition with little research into the underlying mechanism, but felt to be most commonly related to abnormalities of brain neurotransmitters.

**Table 1:** Schedule of Procedures

Procedure	Entry (D0)	Daily 1–5 (on treatment)	Day 6	Final Visit (Day 7)
Consent	○			
Age	○			
Gender	○			
Electrolytes	○			
Liver function	○			
MMSE	○	○	○	○
Digit Span	○	○	○	○
CAM	○	○	○	○
MDAS	○	○	○	○
Motor type	○			
CMI	○			
IQCODE	○^a^			
GDS-15	○			
Katz ADL	○			
Presence of Restraints		○	○	○
# Falls		○	○	○
# Pressure areas	^b^	○	○	○
Rescue medication		○	○	○
Adverse events			○	○
Review INR	○^c^	○^c^		

^a^Ideally the IQCODE will be measured at entry but can be measured at any point

^b^Initial number and location will be recorded at entry in order to derive the number of new areas at each subsequent visit

^c^If participant is on warfarin, we will review the INR that has been performed every 1–2 days as is standard protocol for acutely unwell inpatients when warfarinised

Delirium is a common clinical phenotype with a variety of potential precipitating factors on the background of predisposing factors. The clinical phenotype does not appear to vary with cause [2], and therefore research into the treatment of delirium can be conducted without reference to aetiology. An exception is Delirium Tremens, a specific syndrome caused by withdrawal from chronic alcohol consumption. This is regarded separately from delirium and is not the topic of the proposed study.

One common feature of delirium is a disturbance of the sleep wake cycle [2]. In support of a potential role of melatonin and disturbance of melatonin metabolism in delirium, Balan et al. demonstrated that levels of urinary metabolites of melatonin were altered in delirium, in coordination with the level of psychomotor activity (low in those with hyperactive delirium, high in hypoactive delirium) [3]. Other studies have shown abnormalities of tryptophan (from which melatonin is formed) are associated with postoperative and critical care delirium [4, 5]. Further, case reports of successful melatonin use for the treatment of delirium have been published [6].

The hypothesis that melatonin might be useful for the prevention and treatment of delirium has been tested, with two trials being particularly relevant. Al-Aama et al. randomised 145 recently hospitalised participants in a medical service to melatonin 0.5 mg nocte or placebo for 14 nights or discharge [7]. Delirium diagnosis was assessed with the validated Confusion Assessment Method tool and severity by the validated Memorial Delirium Assessment Scale (MDAS). Delirium was clinically and statistically significantly reduced in the active treatment group by approximately 61 %, by 81 % when incident (pre-existing prior to enrolment) delirium was excluded, and 81 % when dementia and comorbidities were controlled for. By comparison, no medication has been shown to prevent delirium occurrence, and two trials of non-pharmacological treatments showed reductions in the order of 33 % [7]. Delirium severity (on MDAS) was not significantly different, and surrogate outcomes that would suggest efficacy in treatment of delirium such as length of stay, restraints and use of sedatives were also not significantly different.

A second trial examined melatonin 5 mg in prevention of delirium in elderly patients undergoing hip arthroplasty and spinal anaesthesia. 222 patients were randomised to standard treatment, midazolam prior to and post-surgery, or melatonin 5 mg prior to and post-surgery [8]. Delirium was assessed based on decline on the Abbreviated Mental Test from baseline. This methodology has been shown [8] to be a sensitive but not specific method of delirium detection, and likely inferior to the CAM. Delirium occurred in 16/49 control group participants, and 5/53 melatonin group participants, a 72 % relative risk reduction. Although the paper stated melatonin 5 mg was effective in the treatment of delirium, it was not clear how it was evaluated, and personal communication with the author revealed this statement was based on the clinical impression of psychiatrists assisting in the management of delirious patients.

In contrast to these two trials, a recently completed randomised-controlled trial of melatonin 3 mg in the prevention of delirium after hip fracture did not show a significant reduction in the incidence of delirium, nor in the severity of incident delirium [9].

Based on current knowledge, it is plausible that melatonin is useful for the prevention of delirium in elderly patients, that low dose melatonin does not appear to be useful for treatment of delirium, and that high dose melatonin (5 mg) may be effective in the treatment of delirium. However, this needs to be examined further before it can be recommended.

When considering the efficacy of melatonin in the treatment (rather than prophylaxis) of delirium, it should be noted that delirium abatement is regarded purely clinically. Were this decrease or cessation of symptoms to occur after cessation of an effective treatment, compared to standard care, it would constitute evidence of cure. This could be assessed by following trial participants after cessation of active and placebo treatment.

This paper describes the design of the study, “Moderate dose melatonin for the abatement and treatment of delirium in elderly general medical inpatients”, a trial currently in progress.

## Methods and design

**Fig. 1:**
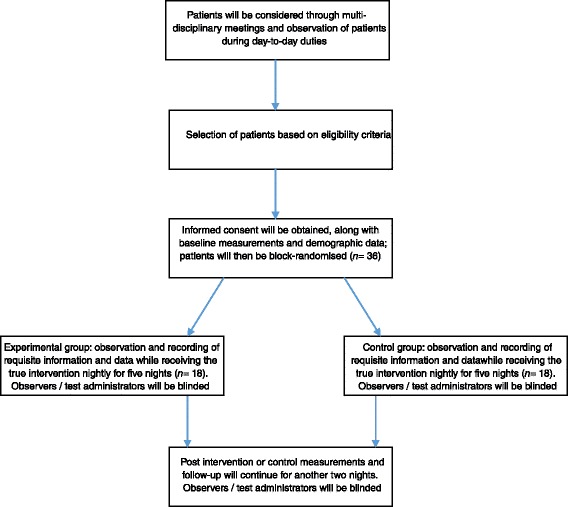
Overview of the study protocol

This trial was designed based on CONSORT guidelines [10]. For a schematic detailing the layout of the study, see Fig. 1. Trial procedures are described in Table 1.

### Research questions

#### Primary research question

1. To determine if melatonin of 5 mg nightly for 5 nights decreases the severity of symptoms of delirium in elderly medical inpatients by evaluation of the MDAS

#### Secondary research questions

1. Abatement of symptoms that continues for 2 further days after the 5 days of treatment
2. The severity and duration of delirium
3. Reduces falls
4. Reduces pressure areas
5. Reduces the use of rescue medications (antipsychotics, benzodiazepines)
6. Improves sleep quality as evaluated in Item 10 of the MDAS
7. Reduces the presence of restraints

### End points

#### Primary

1. Change in MDAS score from baseline to average over days 1–5

#### Secondary

1. Change in MDAS score from average over days 1–5 to average over days 6–7
2. Presence or absence of delirium from days 1–7 as assessed by CAM criteria
3. Number of falls over days 1–5 of the study period
4. Number of pressure areas developing over days 1–5 of the study period
5. Number and dose of rescue medications used over days 1–5 of the study period
6. Change in score on item 10 of the MDAS
7. Number of instances of the use of restraints over days 1–5 of the study period

### Location and setting

The Royal Melbourne Hospital in Parkville, Australia. It is a major teaching hospital with over 60,000 emergency attendances and over 85,000 patient admissions a year into 330 acute beds [11].

### Study population and recruitment

During multidisciplinary meetings and the day-to-day duties of investigators, potential patients will be considered for inclusion. Patients with a diagnosis of delirium via the CAM and who are 70 years or older as general medical inpatients will be screened according to exclusion criteria. These exclusion criteria include solely hypoactive delirium (MDAS question 9), planned surgery, the inability to speak or understand English, severe sensory impairment or dysphasia, an expected prognosis or discharge of less than 7 days, severe hepatic failure (defined as Bilirubin ≥2.5 times upper limit of normal; alkaline phosphatase, aspartate transaminase and/or alanine transaminase > 3 times upper limit of normal), a markedly non-therapeutic INR (<1 or >4), use of melatonin or melatonin receptor agonist in the preceding 14 days, active seizure disorder (a seizure in the last month, or a seizure disorder not on anticonvulsants), and concomitant cimetidine use. As melatonin is a sedative medication, it’s clinically inappropriate to provide this to patients who have an exclusively hypoactive delirium as this may exacerbate the decreased conscious state. Also, as per Balan et al. [3], an exclusively hypoactive delirium may be associated with an elevated melatonin level. Patients who have surgery planned during the study period are to be excluded as surgery and anaesthesia are independent risk factors for delirium and would act as intra-intervention confounders.

Given patients with delirium are unable to provide informed consent, consent will be obtained by the person responsible for surrogate decision making and documented on clear consent forms. The surrogate decision maker retains the capacity to withdraw the patient from the trial at any time. If during the course of the trial the patient regains capacity due to the resolution of their delirium, a separate informed consent will be obtained should the patient wish to continue; if they do not, they will be withdrawn from the trial.

The Melbourne Health Human Research and Ethics Committee has evaluated and approved this trial.

### Randomisation & blinding

After enrolment, patients will be randomised into treatment groups by drawing a sealed, opaque envelope from a box containing an allocation code (randomised by an unrelated statistician). The box contains equal numbers of allocation codes to a total of the expected number of participants. The envelope and code are discarded after being drawn. The envelopes will be in permuted blocks of 4 (2 placebo 2 active).

The results of this randomisation are not known to any of the investigators or intervention administration staff. A separate code interpretation sheet is kept locked in the trial pharmacy, where after the completion of data collection the allocation codes will be matched to provide the requisite placebo vs intervention information.

### Study medication and administration

Study medication tablets appear identical and are packaged in HDPE containers of 5 capsules each. They are prepared in accordance with Good Manufacturing Procedure (GMP) guidelines. On prescription by Medically qualified research staff, a container may be collected from the trial pharmacy upon inclusion of a patient. The medication was purchased through an unrestricted grant from Healthe Care Australia Pty Ltd. The medication is prescribed and administered as per the regular drug chart protocol at our institution, where it is marked as ‘nocte’. As such, to mirror normal practice, it is administered at approximately 8 pm nightly for five nights.

### Data collection

Baseline demographic data and current medication use (including as needed [PRN]) medications will be recorded. Following enrolment, patients will be visited by trained and qualified research staff every day to collect data and assess safety.

Delirium will be diagnosed using the Confusion Assessment Method (CAM) [12], as the primary outcome variable. This is a widely used instrument for the detection of delirium in the acute hospital setting. It has a sensitivity of 94–100 % and a specificity of 90–95 % and generates a DSM IV diagnosis of delirium. Duration of delirium will be recorded in days and delirium considered to have ceased the day of the last visit at which the participant was positive on CAM.

Delirium severity will be measured by the Memorial Delirium Assessment Scale (MDAS) [13], a validated scale suitable for use by both skilled clinical and trained observers, with good internal consistency and inter-rater reliability. The maximum MDAS score will be used as a measure of severity.

Comorbidity will be measured by the Charlson scale (Charlson Comorbidity Index, CMI) [14] from the history, patient, and/or surrogate; pre-morbid function will be measured using the Katz Activities of Daily Living (ADL) [15] index score from their condition two weeks prior to admission; depression will be measured using the GDS-15 item scale [16]; cognition will be measured by the mini-mental state examination (MMSE) [17].

Premorbid cognitive impairment will be determined both from chart review for a diagnosis of dementia or other cause of chronic cognitive impairment by a specialist neurologist, psychiatrist, or geriatrician, and the administration of the Informant Questionnaire for Cognitive Impairment or Dementia in the Elderly short form (IQCODE-sf) [18]. The IQCODE-sf will be completed with the assistance of primary caregivers.

Motor subtype of delirium does not have a validated scale and will be assessed by clinical judgement of the investigators.

Use of rescue medication will be determined from chart review and recorded by medication and average daily dose since last visit, and will be sub-stratified as required into groupings of those with significant anticholinergic activity, opioids, antipsychotics, and benzodiazepines.

### Delirium treatment

All patients will receive care based on current practice at the Royal Melbourne Hospital, which is based on standard procedures including behavioural management, reorientation, and pharmacotherapy when indicated. If a patient is already on antipsychotic medication, this will continue throughout the trial.

### Power analysis

The primary endpoint is a decrease in MDAS score from the baseline to the average score across days 1–5 of treatment. As a pilot trial, one of the feasibility objectives is to obtain the standard deviation for the primary outcome measure in order to allow a power calculation to be performed to assist with the development of an appropriately powered trial to test the relevant hypotheses. As such, no true power calculation can be calculated.

However, a trial of the prophylactic use of melatonin in a Canadian hospital [7] yielded entry data of an MDAS score of 11.4 with a standard deviation (SD) of 3.0. A clinically significant change on the MDAS (highest possible score of 30, lowest of 0, positively correlated with severity) of 3 points at 80 % power and a significance level of 5 % suggests 16 patients per arm. At a 10 % dropout rate and rounding up, the groups should be of 18 patients each.

It should be noted that these figures cannot be used to precisely calculate power, as they use MDAS score at entry into the trial (not during treatment), and the population is significantly different with an average length of stay of 14 days (around twice that of similar patients at RMH). While these results provide some guidance that a trial is possible and relevant group size and power achievable, a pilot is still required to confirm the exact numbers needed.

### Statistics

Data will be collected for each patient on a daily basis, up to 7 days following recruitment. The primary and secondary outcome measures will be compared using standard statistical methodology. For continuous variables, results will be compared using a Students’ t-test with significance set to 0.05 if they are normally distributed, else a Mann-Whitney or other non-parametric test will be utilised. Control and treatment group homogeneity will be tested with Chi-square statistics. For discrete outcomes, results will be tested using a Chi-square test. Risk ratios will be employed where appropriate. The Hausman specification test will be used to determine whether the data should be analysed using either fixed or random effects, though it is expected that a random and fixed (mixed) effects model will be required.

Extensions to the statistical methods above may be required depending on what is found once the data is collected and the analysis is begun.

Missing data will be accounted for using the last observation carried forward method, and all data will be analysed according to the intention-to-treat principle primarily (and per-protocol analysis secondarily).

## Discussion

A large quantity of both scientific and journalistic literature has been generated about the increased number of older persons living worldwide. The risk of developing delirium increases as one ages. Given that delirium has significant health costs to patients, as well as being a significant financial burden to families and to society, an effective treatment for delirium would mitigate against these costs – and no such treatment currently exists. A pilot RCT was selected to appropriately assess the relevant outcome measures as discussed above, and to generate data for powering a larger study in the future. Acute general medical inpatients as a population are at significant risk for developing delirium, and are among a group that we expect to see significant benefit in and for should delirium treatment/cessation be successful. This, in conjunction with not excluding patients with dementia, should allow for greater external validity of the results, and also informs the practice for a large proportion of hospital inpatients.

Our work should allow for further information as to whether melatonin is able to provide therapeutic as well as prophylactic benefit, and as a pilot also guide the powering of further larger trials if they are warranted in the future.
